# Parp mutations protect from mitochondrial toxicity in Alzheimer’s disease

**DOI:** 10.1038/s41419-021-03926-y

**Published:** 2021-06-25

**Authors:** Yizhou Yu, Giorgio Fedele, Ivana Celardo, Samantha H. Y. Loh, L. Miguel Martins

**Affiliations:** MRC Toxicology Unit, University of Cambridge, Cambridge, UK

**Keywords:** Metabolomics, Cell death in the nervous system, Alzheimer's disease

## Abstract

Alzheimer’s disease is the most common age-related neurodegenerative disorder. Familial forms of Alzheimer’s disease associated with the accumulation of a toxic form of amyloid-β (Aβ) peptides are linked to mitochondrial impairment. The coenzyme nicotinamide adenine dinucleotide (NAD^+^) is essential for both mitochondrial bioenergetics and nuclear DNA repair through NAD^+^-consuming poly (ADP-ribose) polymerases (PARPs). Here we analysed the metabolomic changes in flies overexpressing Aβ and showed a decrease of metabolites associated with nicotinate and nicotinamide metabolism, which is critical for mitochondrial function in neurons. We show that increasing the bioavailability of NAD^+^ protects against Aβ toxicity. Pharmacological supplementation using NAM, a form of vitamin B that acts as a precursor for NAD^+^ or a genetic mutation of PARP rescues mitochondrial defects, protects neurons against degeneration and reduces behavioural impairments in a fly model of Alzheimer’s disease. Next, we looked at links between PARP polymorphisms and vitamin B intake in patients with Alzheimer’s disease. We show that polymorphisms in the human *PARP1* gene or the intake of vitamin B are associated with a decrease in the risk and severity of Alzheimer’s disease. We suggest that enhancing the availability of NAD^+^ by either vitamin B supplements or the inhibition of NAD^+^-dependent enzymes such as PARPs are potential therapies for Alzheimer’s disease.

## Introduction

Alzheimer’s disease (AD) is the most common cause of dementia and a major risk factor for developing other diseases [1–4]. There is no cure for AD. Hence, preventive strategies are urgently needed. Familial AD is associated with the accumulation of a toxic form of the amyloid-β (Aβ) peptide in the brain [5–8]. Animal models of AD in the fruit fly *Drosophila melanogaster* were generated by expressing toxic human Aβ in the fly’s neurons [9]. The neuronal expression of the disease-associated Aβ (1-42), with an Arctic mutation (Glu22Gly) (Aβ-Arc), resulted in its accumulation in the neuronal cells. The accumulation of Aβ-Arc in turn causes neurodegeneration and premature neuronal cell death, recapitulating pathological features of AD in humans (reviewed in [10]). Flies are therefore a powerful animal model for studying the mechanisms of neurodegeneration and the identification of novel preventive strategies for this disease [11].

Mitochondria are the cell’s powerhouses. They produce adenosine triphosphate (ATP) as the cellular energy currency. Their compromise has serious consequences for animal tissues with high energetic demands, such as the brain and muscle. The prevailing consensus is that, in AD, mitochondrial impairment is an epiphenomenon of a dysfunctional neuron, secondary to toxic molecular-initiating events (reviewed in [12]).

Nicotinamide adenine dinucleotide (NAD^+^) is important for the generation of ATP and the maintenance of redox balance in the mitochondria. In addition, NAD^+^ is also essential for DNA repair mechanisms in the cell nucleus involving the NAD^+^-consuming enzyme, poly (ADP-ribose) polymerase (PARP). The levels of NAD^+^ are governed by the rates of biosynthesis, consumption, recycling or degradation of this metabolite (reviewed in [13]). In models of mitochondrial dysfunction, increasing the bioavailability of NAD^+^ improves mitochondrial function, enhances oxidative metabolism and general fitness [13]. The inhibition of major NAD^+^-consuming enzymes, such as PARP, or the dietary supplementation of its precursor increases NAD^+^ levels, protects against mitochondrial dysfunction and improves lifespan in fly models of neurodegeneration linked to Parkinson’s disease [14, 15].

Here we explore the metabolic consequences of the expression of toxic Aβ-Arc in flies. We find that metabolites involved in nicotinamide (NAM) metabolism, in particular NAD^+^, are altered. We show that the dietary supplementation of an NAD^+^ precursor vitamin, NAM (or vitamin B_3_), and the genetic suppression of the NAD^+^ consuming enzyme, Parp, improves mitochondrial function and prevents neurodegeneration in flies expressing Aβ-Arc in neurons. We then sought to validate the relationship between NAM, PARP and AD in humans using data from the UK Biobank. We identified three variants in the *PARP1* gene associated with AD and show that participants taking vitamin B, which contains NAM, have lower risk and severity of AD. Our results highlight an important role of mitochondria in AD and the potential for NAD^+^-based therapies.

## Results

### Identification of a metabolic signature linked to nicotinate and NAM metabolism in flies expressing toxic Aβ

**Fig. 1:**
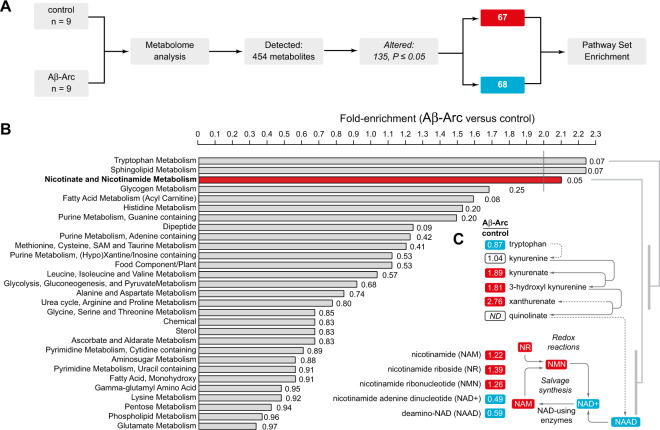
Expression of a toxic form of Aβ results in alterations in tryptophan and nucleotide metabolism. **A** Workflow employed for the identification of metabolic changes in adult flies expressing toxic Aβ-Arc. Significance was determined using Welch’s two-sample *t* test (*n* indicated for each sample set). **B** Pathway enrichment analysis of all significantly altered metabolites. **C** Expression of Aβ-Arc causes changes in tryptophan and NAD metabolism. Red and blue correspond to metabolites that are, respectively, significantly upregulated or downregulated. ND corresponds to a metabolite below detection threshold. Genotypes: control, *da*Gal4>+; Aβ-Arc, *da*Gal4>Aβ-Arc. See also Supplementary Table 1.

To study the metabolic alterations caused by the expression of toxic Aβ in an in vivo model, we conducted a global analysis of the metabolome of adult flies expressing a secreted form of Aβ-Arc [9] in neurons. We found that approximately 30% of the biochemicals measured in whole flies are significantly altered by neuronal expression of Aβ-Arc (Fig. 1A and Supplementary Table 1).

**Fig. 2:**
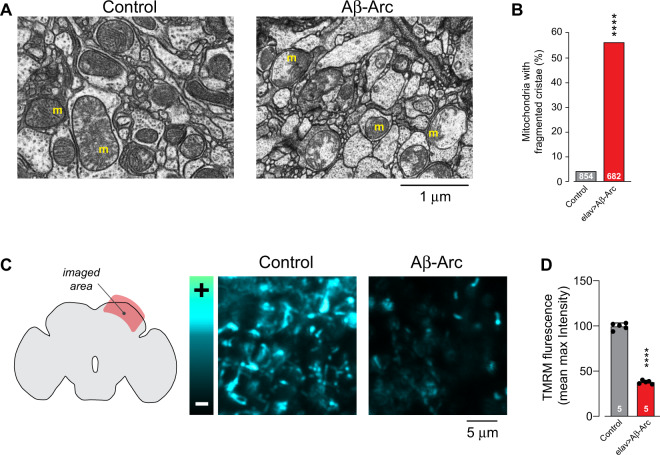
Expression of toxic Aβ causes mitochondrial toxicity. **A** Ultrastructural analysis of adult brains in control (*elav*Gal4>+) and Aβ-Arc-expressing flies (*elav*Gal4>Aβ-Arc) showing mitochondria with fragmented cristae in neuropiles (m, mitochondria). Represented TEM micrographs of the indicated genotypes are shown. **B** Percentages of mitochondria in the neuropile that exhibit fragmented cristae (asterisks, two-tailed chi-square test, 95% confidence interval). **C** Loss of Δψm in the brain mitochondria of flies expressing toxic Aβ-Arc. Representative confocal images of whole mounted brains showing neurons loaded with TMRM are shown. The intensity levels of TMRM are visualised using a five-tone heat map. **D** Expression of toxic Aβ-Arc causes a loss of Δψm in *Drosophila* neurons (mean ± SD; asterisks, two-tailed unpaired *t* test).

Next, we performed a pathway analysis of the biochemicals that were altered in Aβ-Arc flies. We found that Aβ-Arc causes significant alterations in the nicotinate and NAM metabolism pathway, an upregulation in several precursors of the coenzyme NAD^+^ belonging to the tryptophan metabolism pathway and a decrease in NAD^+^, which is essential for generating energy in mitochondria (Fig. 1B, C). Mitochondrial dysfunction is often described in models of AD but the current view is that it represents a secondary feature of a damaged neuron (reviewed in [12]). We therefore tested whether the decrease in NAD^+^ levels observed in adult flies expressing Aβ-Arc was associated with mitochondrial dysfunction in the brain. An ultrastructural analysis by transmission electron microscopy (TEM) of mitochondria in the adult flies’ brain expressing this toxic form of Aβ showed defects in mitochondrial morphology (Fig. 2A, B). Aβ-Arc expressing flies showed a loss of mitochondrial membrane potential (Δψm), indicating a compromise in mitochondrial function (Fig. 2C, D). These findings demonstrate that the mitochondrial impairment caused by neuronal expression of toxic Aβ in flies is linked to decreased NAD^+^ metabolism.

### A NAM-supplemented diet suppresses mitochondrial dysfunction and neurodegeneration in flies expressing toxic Aβ

The co-enzyme NAD^+^ is an essential component of the mitochondrial electron transport chain. NAD^+^ is made from NAM, the amide form of nicotinic acid, also known as niacin or vitamin B_3_ (Fig. 1C). Hence, we tested the effects of enhancing NAD^+^ in flies expressing toxic Aβ with a diet supplemented with NAM.

**Fig. 3:**
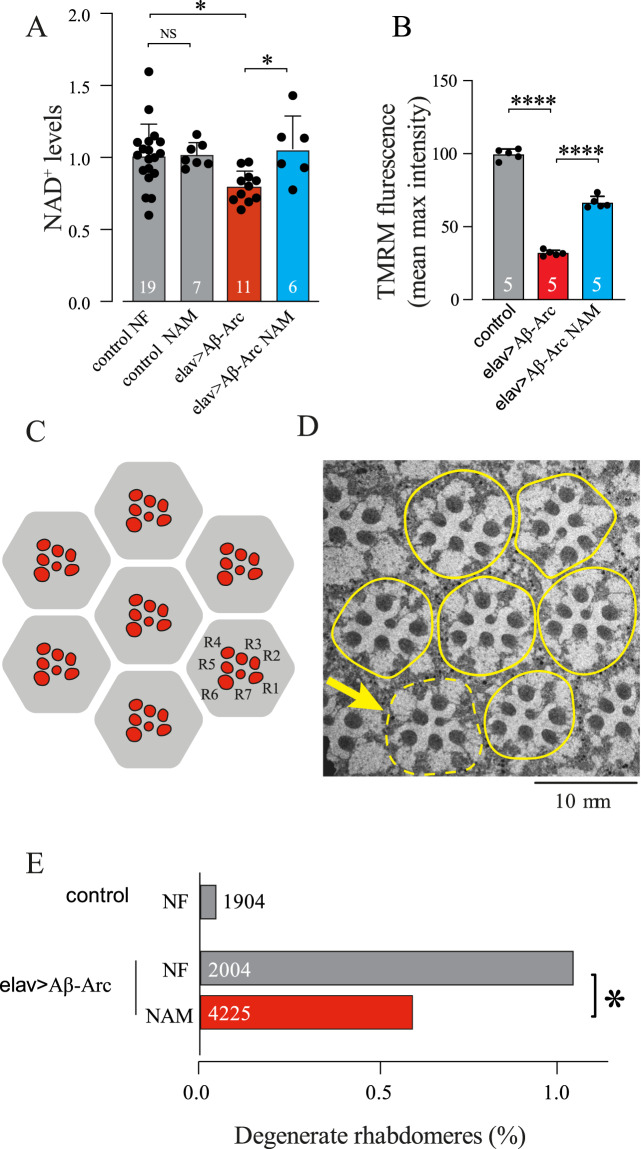
Diet interventions to improve nicotinate and nicotinamide metabolism reduce neurodegeneration in flies expressing toxic Aβ. **A** The NAD^+^ levels of Aβ-expressing flies fed with the NAD^+^ precursor NAM are higher than those fed with normal food (mean ± SD; asterisks, one-way ANOVA with Tukey’s multiple comparison test). Male flies were fed with food containing NAM post eclosion and sacrificed after day 15. **B** Pharmacological supplementation of NAM rescued Δψm in the brains of 5-day-old male flies (mean ± SD; asterisks, one-way ANOVA with Dunnett’s multiple comparison test). **C** An illustration of the typical layout of the visible photoreceptors (red, R1–R7) at the surface of the adult *Drosophila* ommatidia (grey hexagon). **D** Transmission electron micrograph showing individual ommatidia (yellow outlines) in the eye of Aβ-Arc-expressing flies. Arrow points to an ommatidium where a photoreceptor neuron is missing. **E** Dietary supplementation with NAM (5 mM) rescues the neurodegeneration of photoreceptor cells in Aβ-Arc-expressing flies (asterisks, two-tailed chi-square test, 95% confidence interval). NF normal food. Genotypes: control, *elav*Gal4>+; elav>Aβ-Arc, *elav*Gal4>Aβ-Arc.

Flies expressing Aβ-Arc exposed to a NAM-supplemented diet showed an increase in their NAD^+^ pools (Fig. 3A). The dietary supplementation with NAM enhanced Δψm in neuronal mitochondria of flies expressing Aβ-Arc (Fig. 3B), indicating that a NAM-supplemented diet improves mitochondrial function. Expression of toxic Aβ causes age-dependent neurodegeneration in retinal photoreceptor cells. We next tested the effect of a NAM-supplemented diet on the degeneration of retinal photoreceptors of Aβ-Arc-expressing flies. We showed that flies kept on a NAM-supplemented data showed a significant decrease in photoreceptor neurodegeneration (Fig. 3C–E). We conclude that a dietary supplementation with this formulation of vitamin B_3_ improves mitochondrial function and neuronal health in flies expressing toxic Aβ.

### A *Parp* mutation improves mitochondrial function and restores neuronal health in Aβ-Arc-expressing flies

**Fig. 4:**
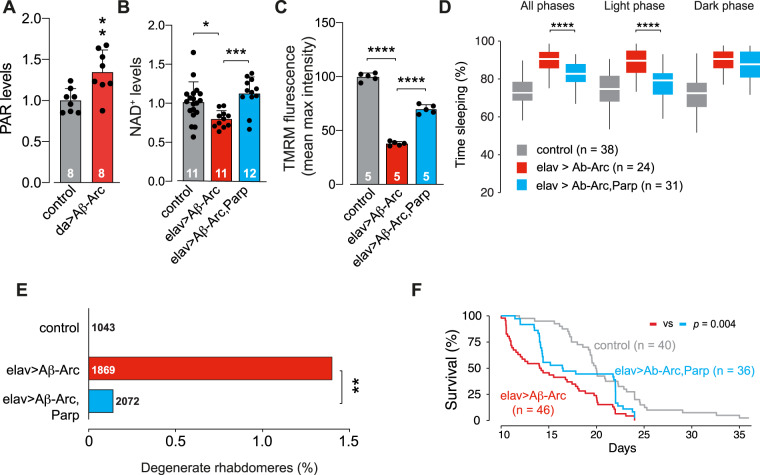
Mutation of *Parp* rescues mitochondrial toxicity associated with the expression of Aβ-Arc. **A** Flies overexpressing Aβ-Arc have higher levels of poly ADP-ribosylation (mean ± SD; asterisks, two-tailed unpaired *t* test). **B** A *Parp* mutation increases NAD^+^ levels in Aβ-Arc-expressing flies (mean ± SD; asterisks, one-way ANOVA with Dunnett’s multiple comparison test). **C** A *Parp* mutation rescues the loss of Δψm in the brains of Aβ-Arc-expressing flies (means ± SD; asterisks, two-way ANOVA with Dunnett’s multiple comparison test). **D** A *Parp* mutation rescues the total percentage of time spent asleep, as well as sleep during the light phase (asterisks, one-way ANOVA with Tukey’s multiple comparison test for All phases and Light phase). **E** A *Parp* mutation rescues the neurodegeneration of photoreceptor cells in Aβ-Arc-expressing flies (asterisks, two-tailed chi-square test, 95% confidence interval). **F** A *Parp* mutation improves the lifespan of Aβ-Arc-expressing flies and significances were determined using the log-rank, Mantel–Cox test. Genotypes: control, *da*Gal4>+ (**A**), *elav*Gal4>+ (**B**–**F**); da>Aβ-Arc*, da*Gal4>Aβ-Arc (**A**), elav>Aβ -Arc, *elav*Gal4>Aβ-Arc (**B**–**F**).

The coenzyme NAD^+^ is not only essential for energy metabolism but also acts as a co-substrate in biochemical reactions such as protein ADP-ribosylation (PAR), catalysed by PARPs, reviewed in [16]. We have previously shown that mitochondrial dysfunction in *pink1* or *parkin* mutant flies, two genes implicated in Parkinson’s disease, causes an increase in PAR levels. Introducing a *Parp* mutation in either *pink1* or *parkin* mutants boosted NAD^+^ levels and protected dopaminergic neurons loss. As we also detected a loss of mitochondrial function (Fig. 2C, D) in Aβ-Arc-expressing flies, we compared the PAR levels between control and Aβ-Arc-expressing flies. We found that the expression of Aβ-Arc led to an increase in PAR in whole flies (Fig. 4A). Next, we introduced a *Parp* mutation in flies expressing toxic Aβ to test attempt to boost NAD^+^ levels. Neuronal expression of toxic Aβ in flies with a *Parp* mutation (Parp^CH1^/+) resulted in an increase in NAD^+^ levels (Fig. 4B) and improved mitochondrial function (Fig. 4C).

In flies, Aβ toxicity influences neuronal activity and disrupts sleep [17]. We observed that, in the presence of the *Parp* mutation, flies expressing toxic Aβ showed an improvement of their sleep patterns (Fig. 4D). Moreover, *Parp* mutation also reduced neurodegeneration (Fig. 4E) and improved the mid-life survival of Aβ-Arc-expressing flies (Fig. 4F). We conclude that a mutation in the *Parp* gene in flies expressing toxic Aβ rescues mitochondrial dysfunction and is neuroprotective.

### Genetic variants in human *PARP1* predict the risk of AD

**Fig. 5:**
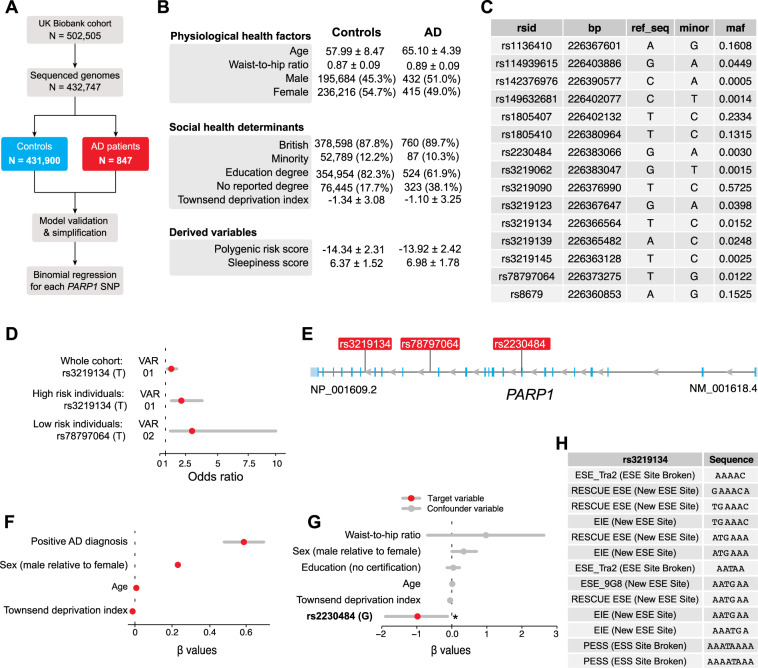
Gene variants in human *PARP1* alter the risk and severity of AD. **A** Workflow of the analysis. Out of a total of 502,505 UK Biobank participants, 432,747 patients who contained genomic data were analysed. **B** Descriptive statistics of the UK Biobank cohort that was analysed. Either the number of participants in each category and their percentage with respect to the total cohort or the mean and standard deviation are shown. A full table of summary characteristics is available in Supplementary Table 3. **C** Table of the 15 SNPs in *PARP1* present in our cohort. The SNP name (rsid), base position (bp), reference sequence (ref_seq), minor allele (minor) and minor allele frequencies are shown. This information was extracted from the NCBI database. **D** Odds ratios of the significant SNP in *PARP1* associated with the risk of AD and their respective 95% confidence intervals. The odds ratios are calculated with respect to the allele shown in parentheses using individual binomial models that accounts for covariates. Only the significant SNPs are shown. Statistics of all 15 SNPs analysed are shown in Supplementary Table 4. **E** Genomic location of the *PARP1* SNPs that are significantly associated with AD risk (VAR01 and VAR02) as well as sleepiness in AD (VAR03, explained in the next figure). **F** AD patients have a higher risk of sleepiness. *β* values with respect to the sleepiness score are calculated using a linear regression, after accounting for age, sex, social deprivation (Townsend deprivation score), education and obesity. Only the significant associations are shown. The numeric values of this model are in Supplementary Table 7. **G** *β* values of the model containing the significant SNP (rs2230484) that is associated with differences in sleepiness in the subset of patients with AD. Statistics of all 15 SNPs investigated are shown in Supplementary Table 8. **H** Theoretical impact of the SNP rs3219134 (T to C) on the gene-splicing efficiency. ESE Exonic Splicing Enhancers, ESS Exonic Splicing Silencer, EIE exon-identity elements.

We found that a mutation in the *Drosophila Parp* gene protects flies from Aβ toxicity. This led us to test the association between genetic variants in the human *PARP1* gene and the risk of AD. The UK Biobank is a biomedical database and research resource containing health records of more than half a million UK individuals [18]. Out of a total of 502,505 UK Biobank participants, we first identified a subset (432,747) with genomic information (polymorphisms, Supplementary Figs. 1–4 and Supplementary Tables 2 and 3). From this subset, 847 have a positive diagnosis of AD (Fig. 5A, B). Next, we screened for the presence of single-nucleotide polymorphisms (SNPs) in *PARP1* and identified 15 SNPs per individual in our data set (Fig. 5C and Supplementary Figs. 3 and 4). We used an iterative variable selection procedure that combines unsupervised stepwise forward and backward regression analyses [19] and identified main confounding variables, including age, education level, sex and waist-to-hip ratios, that contributed to higher risk of AD. We accounted for them in all subsequent models (Fig. 5B).

We calculated the odds ratios (ORs) of each SNP and AD, which quantifies the increase in likelihood of developing AD associated with having a certain allele. We found that the major allele for the *PARP1* SNP rs3219134 (VAR01) confers a significantly higher risk of AD (OR 1.42; 95% confidence interval (CI) 1.06–1.96, *P* value = 0.03; Fig. 5D, E and Supplementary Table 4). We then performed a sensitivity analysis to generate a polygenic risk score for AD by combining the risk genes identified in two meta-analyses (Supplementary Figs. 3 and 4 and Supplementary Table 5) [20, 21]. We validated that a higher polygenic risk score corresponds to an increased risk of having AD in our cohort (Supplementary Table 6). In the subset of participants with a higher polygenic risk score for AD, we found that the major allele for VAR01 is associated with higher risk of developing AD (OR 2.22; 95% CI 1.38–3.87, *P* value = 0.002; Fig. 5D, E). This indicates that UK Biobank participants with the minor allele for VAR01 are 42% less likely to have a positive AD diagnosis, and this association increases to 222% in participants with a higher polygenic risk score. We identified a different SNP in *PARP1*, rs78797064 (VAR02), in the subset of patients with a lower genetic burden for AD (Fig. 5D, E). The major allele of VAR02 is associated with a higher risk of AD (OR 3.10; 95% CI 1.33–10.00, *P* value = 0.02; Fig. 5D, E).

Disturbed sleep, which occurs in 25–40% of patients with AD [22, 23], impairs the quality of life of AD patients. It precedes other markers like cognitive impairment and the severity of this phenotype increases as the disease progresses [24]. Based on our results which showed that *PARP1* rescues excessive sleep in flies, we investigated whether SNPs in *PARP1* modulate sleep in humans. First, we built a sleepiness score based on three criteria: sleep duration, daytime sleepiness, and the absence of insomnia (see “Curation of the UK Biobank data” in the “Methods” section for further details). Using these scoring criteria, we calculated *β* values, which quantifies the absolute change in the sleepiness score given an exposure variable. We found that having a positive AD diagnosis is the largest contributor of increased sleepiness (*β* value 0.56; 95% CI 0.46 to 0.66, *P* value < 0.0001, Fig. 5F and Supplementary Table 7). Focussing our analysis on patients with AD, we then found that the *PARP1* SNP rs2230484 (VAR03) modulates sleep in AD patients (*β* value −0.98; 95% CI −1.92 to −0.05, *P* value = 0.04; Fig. 5G and Supplementary Table 8). Combined, this data indicates that mutations in *PARP* in both humans and flies affect sleep in AD. VAR03 is located in the exon 8 of the *PARP1* gene (Fig. 5E) and leads to a missense mutation (Pro377Ser) in the linker region after the DNA-binding domain (Zn3) of PARP1. The structure of this region has not been determined [25] so we did not pursue further analyses on VAR03.

VAR01 and VAR02 map to intron 17 and intron 14 of the *PARP1* gene, respectively (Fig. 5E). We therefore asked whether they could affect splicing of the *PARP1* mRNA. To test this, we used Human Splicing Finder [26] to investigate whether these two SNPs have the potential of altering the splicing efficiency of *PARP1*. This analysis indicates that VAR01 is a determinant of splicing efficiency (Fig. 5H). This suggests that the minor allele of VAR01 might confer protective effects against AD by affecting the splicing efficiency of *PARP1*.

### Vitamin B is associated with a lower incidence and severity of AD

**Fig. 6:**
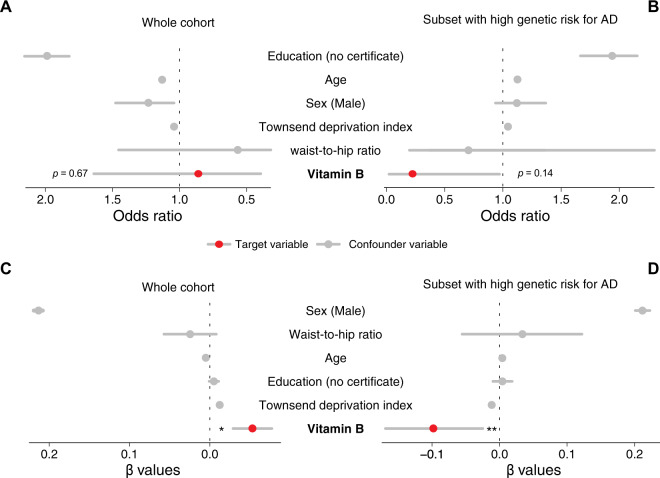
Vitamin B alters the risk and severity of AD. **A** There is a trend for vitamin B being protective against AD in the whole UK Biobank cohort (OR 0.86; 95% CI 0.37 to 1.67, *P* value = 0.67). See Supplementary Table 9 for details. **B** This trend is more robust in participants with a higher genetic risk for AD (OR 0.22; 95% CI 0.01 to 0.99, *P* value = 0.14). See Supplementary Table 9 for details. **C** Vitamin B intake is associated with decreased sleepiness in the whole cohort (*β* value = −0.05; 95% CI −0.10 to −0.005, *P* value = 0.03, see Supplementary Table 11 for details) and **D** individuals with high genetic risk for AD (*β* value = −0.10; 95% CI −0.15 to −0.03, *P* value = 0.005, see Supplementary Table 12 for details).

Based on the observation of the protective effect of SNPs in *PARP1* from the UK Biobank participants, we investigated whether the intake of vitamin B, which contains vitamin B_3_ or NAM, is associated with a lower incidence and severity of AD. In our full data set of 502,505 participants, 3940 participants are described to take vitamin B and 1005 participants have AD. We found that the vitamin B intake is associated with a 16% decrease in the odds of having a positive diagnosis of AD in the whole cohort (OR 0.86; 95% CI 0.37–1.67, *P* value = 0.67), while accounting for education, age, sex, deprivation and obesity as confounding variables (Fig. 6A and Supplementary Table 9). To assess the robustness of this trend, we focussed our analysis on the participants with a higher risk of developing AD as predicted using our polygenic risk score. We found that the protective effect of vitamin B is associated with a 455% decrease in the risk of having AD (OR 0.22; 95% CI 0.01–0.99, *P* value = 0.14; Fig. 6B and Supplementary Table 10).

To further investigate the protective effect of vitamin B, we analysed whether vitamin B decreases sleepiness using our sleepiness score, which we identified as a marker for AD. Vitamin B deficiency causes excess sleepiness [27], and its supplementation might improve vigilance [28]. We show that vitamin B intake is associated with a 0.05 decrease in our sleepiness score (*β* value = −0.05; 95% CI −0.10 to −0.005, *P* value = 0.03; Fig. 6C and Supplementary Table 11). The protective effect of vitamin B against sleepiness is two times stronger in the subset of patients with a higher genetic risk of AD (*β* value −0.10; 95% CI −0.15 to −0.03, *P* value = 0.005; Fig. 6D and Supplementary Table 12).

Taken together, our results show a protective trend for vitamin B against AD as well as a decrease in a characteristic of AD, sleepiness, in participants who take vitamin B.

The strengthening of these associations in the subset of participants with a higher genetic risk reinforce the protective trend for vitamin B.

## Discussion

Deficiencies in NAD^+^ have been reported in age-associated conditions, including cardiovascular, gastrointestinal and neurodegenerative diseases (reviewed in [16]). Here we report a decrease in nicotinate and NAM metabolism and NAD^+^ levels in a fly model of AD. NAD^+^ is synthesised via the de novo biosynthesis pathway and recovered by recycling through the salvage pathway that recycles NAM, the end product of NAD^+^ utilisation in cells. We showed that dietary supplementation of NAM suppresses mitochondrial dysfunction and neurodegeneration. We also show that vitamin B, which encompasses NAM, has protective effects against AD in humans. Our results support a previous study showing a protective effect of niacin, the amide derivative of NAM, against the development of AD and cognitive decline [29]. The salvage pathway is particularly attractive since boosting the availability of NAD^+^ precursors have the potential to delay ageing and a wide range of diseases [30]. Based on the protective effect of NAM in a mouse model of AD, a phase two clinical trial on using NAM as an early AD treatment has been initiated (NCT03061474) in 48 participants.

We then showed that *Parp* mutation alleviates Aβ toxicity in flies and replicated our results in humans by showing that variants in *PARP* protects against AD using data from the UK Biobank. We used Human Splicing Finder to investigate effect of these variants on mRNA processing and found that the minor allele of a novel SNP, VAR01 (rs3219134), may lead to differences in splicing efficiencies, thus leading to a reduction in the total PARP1 protein levels. However, these results are theoretical and require verification experimentally. Our results confirm findings from a study showing that the deletion of CD38, an NAD glycohydrolase, attenuates the pathology of AD by increasing NAD^+^ levels [31]. These results indicate that the suppression of the activity of NAD-consuming enzymes could be a viable approach for neuroprotection in AD. Future studies could probe the effect of genetic mutations in *PARP1* and *CD38* and the dietary intake of vitamin B on mitochondria in patients. Novel positron emission tomography probes can be used to quantify the function of mitochondrial complex 1 non-invasively [32]. They may be used in combination with fluid biomarkers of mitochondrial integrity [33, 34]. In addition, NAD^+^ can also be obtained from other sources, such as bacteria present in the gut microbiota. The presence of commensal bacteria that produce NAM in the intestine of mouse model of amyotrophic lateral sclerosis ameliorated their symptoms [35]. Future work could explore the association between gut bacteria that produce NAM and neurodegenerative diseases.

In summary, we showed, using both genetic and pharmacological approaches, that enhancing nicotinate and NAM metabolism enhances mitochondrial function and results in neuroprotection in a model of AD. Further, we provide evidence for a link between *PARP* mutations and the severity of AD in humans using the UK Biobank.

## Methods

### Genetics and *Drosophila* strains

Fly stocks and crosses were maintained on standard cornmeal agar media at 25 °C. The strains used were *elav*GAL4 and *da*Gal4 (Bloomington Drosophila Stock Center # 458), *w; UAS_Aβ42ARC; +* (a kind gift from D. Crowther, R&D Neuroscience, Innovative Medicines and Early Development Biotech Unit, AstraZeneca, Cambridge, UK) and *w; +; parp*^*CH1*^ (a kind gift from V. Corces, Department of Biology, Emory University, Atlanta, USA).

### Metabolic profiling

Global metabolic profiles were obtained using the Metabolon Platform (Metabolon Inc., NC, USA) as previously described [15]. Whole female flies aged 15 days after eclosion were used. Toxic Aβ42Arc was expressed using the ubiquitous *daughterless* (*da*) Gal4 driver in the whole fly. Each biological replicate comprises 100 15-day-old adult female flies (approximately 100 mg per replicate), with a total of 7–8 replicates per genotype. For sample extraction, a 80% (v/v) methanol:water solution was used. Samples were then prepared for liquid chromatography–mass spectrometry. Compounds above the detection threshold were identified by comparison with library entries of purified standards or recurrent unknown entities. Subsequent analyses were performed using MetaboAnalystR [36]. Undetected metabolites are imputed with the minimum. Identification of known chemical entities was based on comparison with metabolomic library entries of purified standards. Each biochemical is rescaled to set the median equal to 1. Values for each sample are normalised by Bradford protein concentration. The processed values of each metabolite are shown in Supplementary Table 1.

### Drug treatments

NAM was incorporated directly into the fly food at a final concentration of 5 mM. Crosses were set up on normal food and transferred to NAM-containing food after 2 days. Larvae were treated with NAM throughout development. The adult flies were kept on drug-containing food throughout their lifespan, and they were transferred to vials with fresh food every 2–3 days. Flies from each genotype were randomly assigned to normal food and food supplemented with NAM.

### Quantitation of NAD^+^ and PAR levels

NAD^+^ concentrations were measured using the NAD^+^/NADH EnzyChrom colorimetric assay according to the manufacturer’s instructions (BioAssay Systems, CA, USA) as previously described [15]. Briefly, two whole male flies were homogenised in the specified buffer on ice at time ZT-06. NAD^+^ concentrations were normalised to total protein, measured using the bicinchoninic acid assay. The normalised NAD^+^ levels are divided by the average of controls, which were elav>+. Three biological replicates or more were performed for each condition.

PAR levels in flies were determined using the PAR ELISA Kit (Cell Biolabs, XDN-5114). Briefly, 30 whole male flies aged 20 days were used per biological replicate. Samples were frozen using liquid nitrogen and homogenised in radioimmunoprecipitation assay buffer on ice (Thermo Scientific, CAT-89900) containing PARP inhibitors at time ZT-06. The following steps of the assay were performed according to manufacturer’s standard instructions. Absorbance was measured at 450 nm. Assays were conducted on 96-well microtiter plates using an Infinite M200Pro multifunction reader (TECAN, Mannedorf, Switzerland).

### Locomotor assays and lifespan analysis

Adult male flies were aged to 10 days old post eclosion and individually loaded in glass tubes (80 mm × 5 mm × 3 mm) containing the same food used for rearing. The flies were grown and analysed in a light/dark 12 h/12 h cycle at 25 °C. The total number of recorded midline crossings per minute was recorded using the *Drosophila* Activity Monitoring System (Trikinetics, Waltham, MA), and the data were analysed using Rethomics [37]. The analysis started at the first ZT0 to allow acclimation. Sleep was calculated for the first 5 days and the data of flies that died were discarded. Sleep is defined as 5 min of inactivity. One-way analysis of variance with Tukey’s multiple comparison test was used to determine significance for the fraction of time asleep. The data for lifespan analysis are presented as Kaplan–Meier survival distributions. We recorded the entire lifespan of the flies from 10 days post-eclosion until their death and determined statistical significance using the log-rank test. We provide the full analysis script and the raw data in our GitHub repository: https://github.com/M1gus/AD_Parp.

### Pseudopupil analysis

The heads of 4–5-day-old flies were directly fixed on standard microscope slides using quick-dry transparent nail varnish as described [38]. A Zeiss Axioplan 2 microscope equipped with a ×63 oil immersion objective was used to visualise the ommatidia. In all, 5–8 flies per condition were examined, to obtain a total number of around 200 ommatidia or ~1400 rhabdomeres. The percent abnormal rhabdomeres was calculated as the number of degenerate rhabdomeres over the total number of rhabdomeres: (*A* × 1 + *B* × 2 + *C* × 3)/*N*, where *A* = number of ommatidia with 6 rhabdomeres, *B* = number of ommatidia with 5 rhabdomeres, *C* = number of ommatidia with 4 rhabdomeres and *N* = total number of ommatidia counted. Statistical significance was determined using chi-squared test.

### Microscopy-based assessment of mitochondrial function and morphology

Measurements of Δψm in fly brains were performed as previously described [39]. Briefly, fly brains were loaded for 40 min at room temperature with 40 nM TMRM in loading buffer (10 mM HEPES pH 7.35, 156 mM NaCl, 3 mM KCl, 2 mM MgSO_4_, 1.25 mM KH_2_PO_4_, 2 mM CaCl_2_, 10 mM glucose) and the dye was present during the experiment. In these experiments, TMRM is used in the redistribution mode to assess Δψm, and therefore a reduction in TMRM fluorescence represents mitochondrial depolarisation. Confocal images were obtained using a Zeiss LSM 880 confocal microscope equipped with a ×40 oil immersion objective. Illumination intensity was kept to a minimum (at 0.1–0.2% of laser output) to avoid phototoxicity and the pinhole was set to give an optical slice of 2 μm. Fluorescence was quantified by exciting TMRM using the 565 nm laser and measured above 580 nm. *Z*-stacks of 5 fields of 300 μm^2^ each per brain were acquired, and the mean maximal fluorescence intensity was measured for each group.

### Electron microscopy

For TEM, adult fly thoraces were fixed overnight in 0.1 M sodium cacodylate buffer (pH 7.4) containing 2% paraformaldehyde, 2.5% glutaraldehyde and 0.1% Tween-20. Samples were post-fixed for 1 h at room temperature in a solution containing 1% osmium tetroxide and 1% potassium ferrocyanide. After fixation, samples were stained en bloc with 5% aqueous uranyl acetate overnight at room temperature; the samples were then dehydrated via a series of ethanol washes and embedded in TAAB epoxy resin (TAAB Laboratories Equipment Ltd., Aldermaston, UK). Semi-thin sections were stained with toluidine blue, and areas of the sections were selected for ultramicrotomy. Ultrathin sections were stained with lead citrate and imaged using a MegaView 3 digital camera and iTEM software (Olympus Soft Imaging Solutions GmbH, Münster, Germany) in a Jeol 100-CXII electron microscope (Jeol UK Ltd., Welwyn Garden City, UK).

### Statistical analyses

Statistical analyses were performed using GraphPad Prism 9 (www.graphpad.com). The data are presented as the mean values, and the error bars indicate ±SD. The number of biological replicates per experimental variable (*n*) is indicated in either the respective figure or figure legend. No sample was excluded from analysis, unless otherwise stated. Blinding was not done. Significance is indicated as an asterisk (*) for *P* < 0.05, double asterisks (**) for *P* < 0.01, triple asterisks (***) for *P* < 0.001, four asterisks (****) for *P* < 0.0001 and NS for *P* ≥ 0.05. We compiled all biochemical data into a Prism file and uploaded this in our GitHub repository: https://github.com/M1gus/AD_Parp.

### Digital image processing

Fluorescence and TEM images were acquired as uncompressed bitmapped digital data (TIFF format) and processed using Adobe Photoshop, employing established scientific imaging workflows [40].

### UK Biobank data sources

The UK Biobank comprises health data from over 500,000 community volunteers based in England, Scotland and Wales. Information about the geographical regions, recruitment and other characteristics has been previously described [18]. Informed consent was obtained from all subjects. Briefly, between 2006 and 2010, adults aged between 40 and 69 years within close proximity to 1 of the 22 UK Biobank recruitment centres were invited to participate. Individuals had extensive demographic, lifestyle, clinical and radiological information collected. Baseline assessments also included a comprehensive series of questionnaires, face-to-face interviews, physical examinations and blood sampling, with linkages to electronic medical records.

Clinical data on neurodegenerative disorders and other comorbidities were cross-validated by an algorithm from the UK Biobank, which took into consideration the UK Biobank baseline assessment data (verbal interview), linked hospital admissions data and death register data. Specifically, the diagnosis of neurodegenerative diseases and dementia relied on consensus between primary care and hospital admissions and/or mortality data. This method has been previously validated using a subset of the UK Biobank participants and was shown to have high accuracies of detecting true positives [41]. The full protocol is publicly available, and summary data can be viewed on the UK Biobank website (www.ukbiobank.ac.uk).

Vitamin B is a group of eight nutrients. Information on the intake of medications and supplements is obtained through verbal interviews via a touch screen-based questionnaire as detailed in the manual that details the interview procedure at an Assessment Centre of the UK Biobank [42]. To record the use of a medication, the UK Biobank interviewer selects the medication from a comprehensive list of all medications available in Britain. The variable we used for vitamin B intake is labelled as “vitamin b compound tablet”. We then grouped NAM and niacin, which are components of vitamin B, into the vitamin B variable. Dose information was not collected. We excluded specific forms of vitamin B that were unrelated to vitamin B3: vitamin B1, vitamin B6, cantassium vitamin b6, and vitamin b12.

UK Biobank ethical approval was granted from the North West Multi-Centre Research Ethics Committee. The current analysis was approved under the UK Biobank application #60124. A detailed list of the variables of the present study is presented in Supplementary Table 2 and their distributions are shown in Supplementary Figs. 1–4.

### Curation of the UK Biobank data

For the analysis of the relationship between mutations in *PARP1* and AD, we first selected UK Biobank participants whose genome was sequenced. We did not perform any imputation on the genomic data. We then created a sleepiness score by adding sleep duration (in hour) and daytime sleepiness (self-reported score of range 0 to 3) and the absence of sleeplessness or insomnia (self-reported score of range 0 to 2). Details of these variables can be found on the UK Biobank website (https://biobank.ndph.ox.ac.uk/showcase/label.cgi?id=100057).

We used an exploratory approach to select the main comorbidities that influence the risk of AD. The putative comorbidities are presented in Supplementary Table 3. We applied an iterative variable selection procedure combining unsupervised stepwise forward and stepwise backward regression analyses to select the most suitable predictor or combination of predictors in our models based on the Akaike information criterion. These were age, waist-to-hip ratio, sex, ethnicity, education level, social deprivation (Townsend deprivation index) and sleepiness.

We then built a polygenic risk score as a predictor of the genetic burden for AD. We fitted binomial regression models where the response variables were whether a participant had a positive diagnosis for AD against SNPs identified previously [20, 21]. We then created the polygenic risk score by multiplying the OR value of each SNP against the number of the allele present. We validated this score and found that it significantly increases the risk of AD in our cohort (Supplementary Tables 5 and 6).

### Analysis of the UK Biobank data

To determine whether any SNP in *PARP1* identified in our cohort influence the risk of AD, we built individual binomial models iteratively for each *PARP1* SNP, where the response variable was AD and accounting for the covariates identified in the “UK Biobank data curation” section to account for potential linkage effects. We repeated this process for people with a higher and lower genetic burden as determined by our polygenic risk score. Finally, this same workflow was applied to identify any modulators of sleepiness by changing the response variable to our sleepiness score and the type of model to linear regressions.

For each model, we calculated the OR or risk ratio and their 95% CIs to quantify the effects of the independent variables on the response variables. The models were built using the MASS package [19] in R. The comparison tables were generated using the Stargazer package [43]. The analysis source code, detailed quality checks and all supplementary material are available in GitHub (https://github.com/M1gus/AD_Parp).

## Supplementary information

Supplementary Figure 1

Supplementary Figure 2

Supplementary Figure 3

Supplementary Figure 4

Supplementary Table 1

Supplementary Table 2

Supplementary Table 3

Supplementary Table 4

Supplementary Table 5

Supplementary Table 6

Supplementary Table 7

Supplementary Table 8

Supplementary Table 9

Supplementary Table 10

Supplementary Table 11

Supplementary Table 12

## Data Availability

Code generated in this manuscript is deposited in GitHub (https://github.com/M1gus/AD_Parp).
