# Correction: Parp mutations protect from mitochondrial toxicity in Alzheimer’s disease

**DOI:** 10.1038/s41419-021-03976-2

**Published:** 2021-07-20

**Authors:** Yizhou Yu, Giorgio Fedele, Ivana Celardo, Samantha H. Y. Loh, L. Miguel Martins

**Affiliations:** MRC Toxicology Unit, University of Cambridge, Cambridge, UK

**Keywords:** Metabolomics, Cell death in the nervous system, Alzheimer's disease

Correction to: *Cell Death and Disease* 10.1038/s41419-021-03926-y, published online 25 June 2021

The original version of this article unfortunately contained several mistakes in figures:

For Figures 1A and 1C, the text and number in oblongs should be highlighted in red and blue, not red and grey. For Figure 2C, the imaged area should be fully coloured in pink. For Figures 5 and 6 please see the correct figures below. We sincerely apologize for these typeset error. The corrected figures can be found below. The original article has been corrected.

**Figure:**
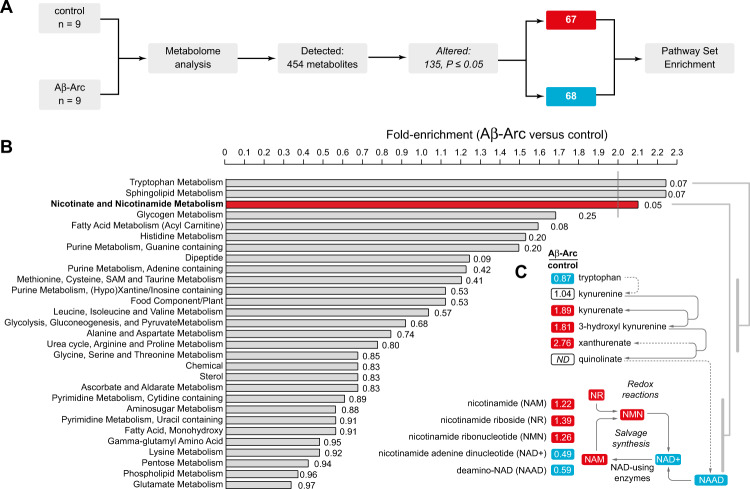
Fig. 1.

**Figure:**
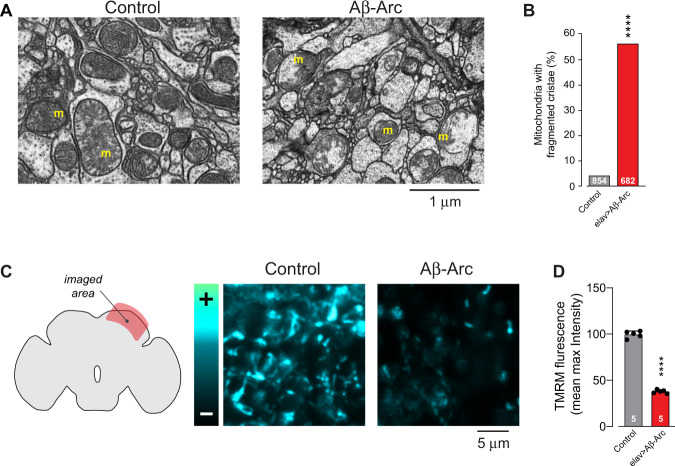
Fig. 2.

**Figure:**
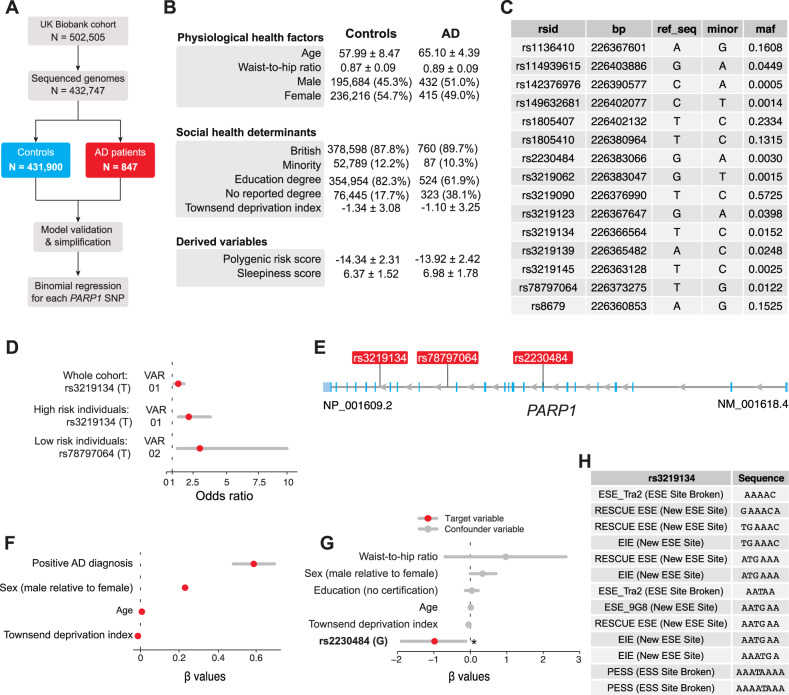
Fig. 5.

**Figure:**
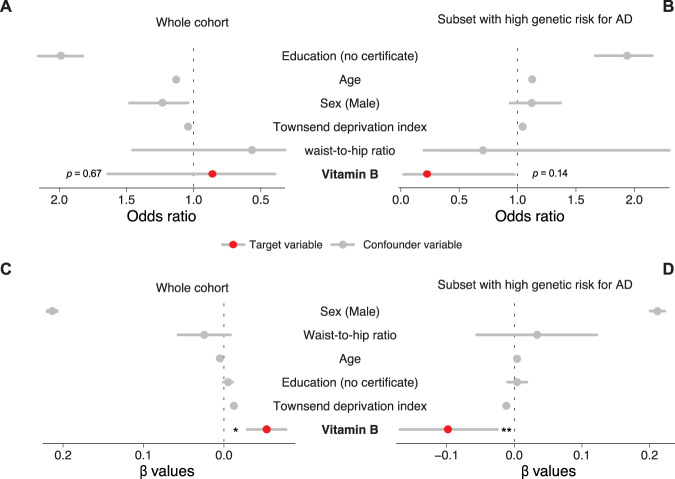
Fig. 6.

