# Supplementary figures and images for: Parp mutations protect from mitochondrial toxicity in Alzheimer’s disease
Source: Cell Death Dis. 2021 Jun 25;12(7):651. doi: 10.1038/s41419-021-03926-y (PMC8233423; doi:10.1038/s41419-021-03926-y)

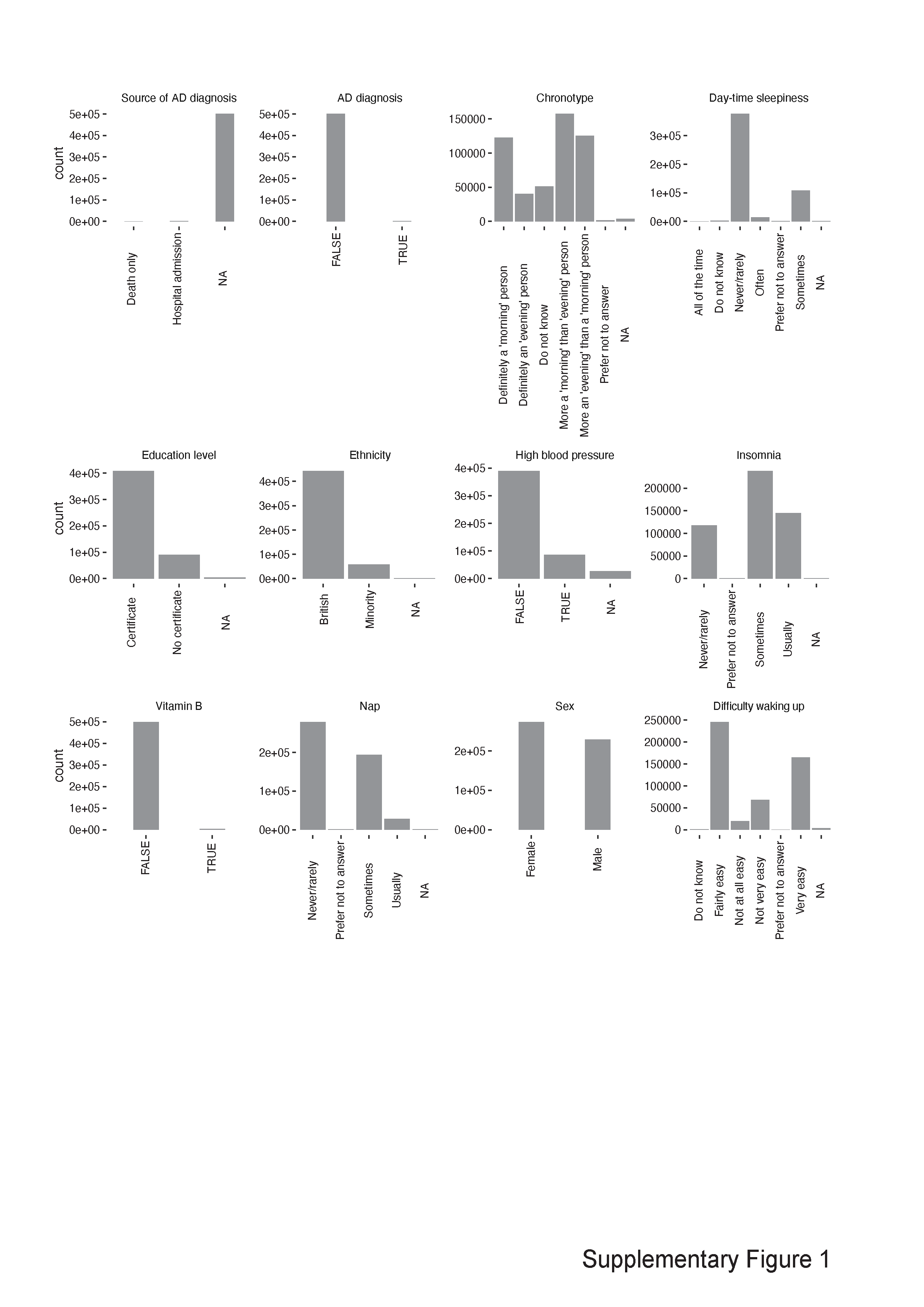

Supplement: Supplementary file 1 — Supplementary Figure 1 [file 41419_2021_3926_MOESM1_ESM.png]

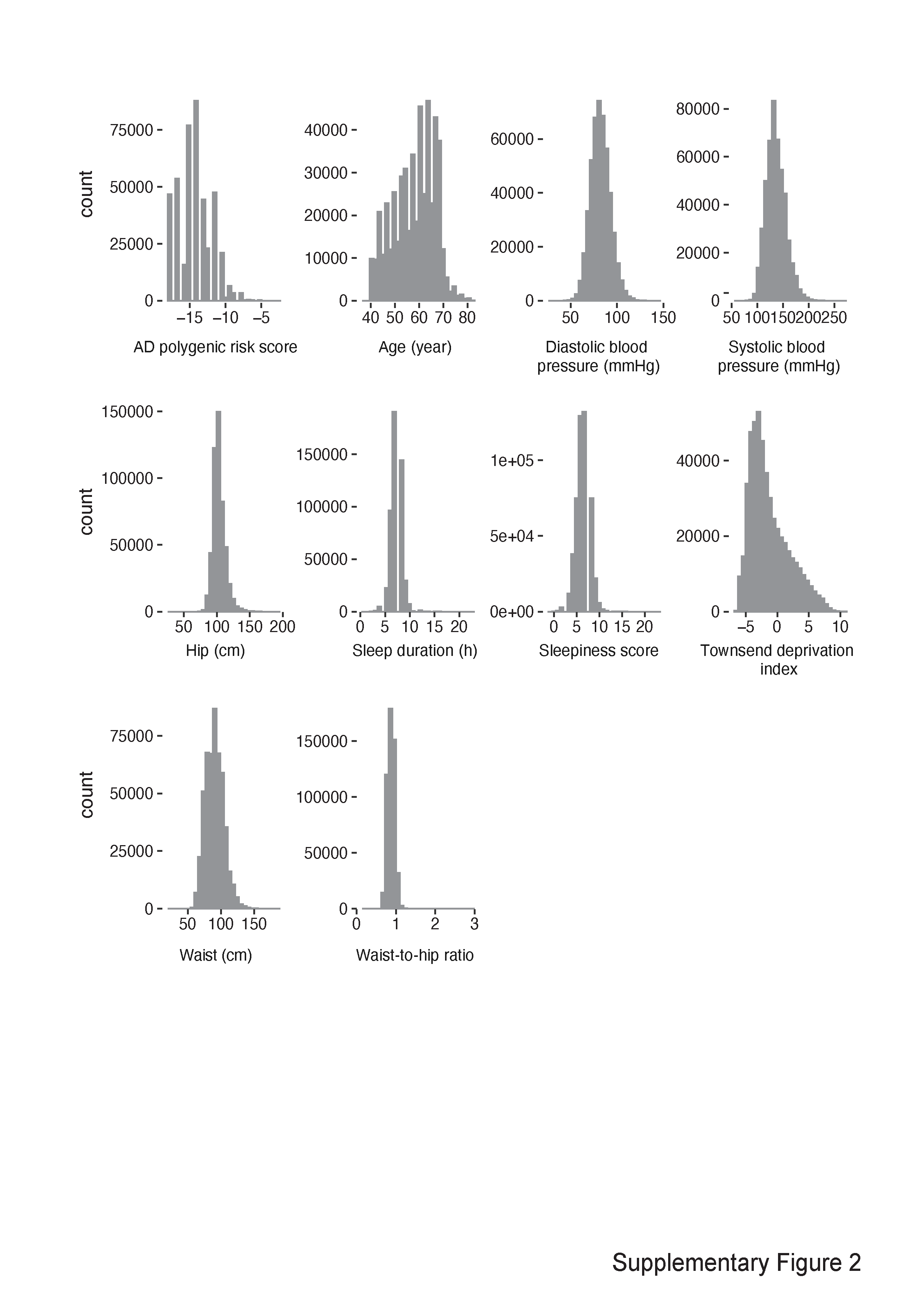

Supplement: Supplementary file 2 — Supplementary Figure 2 [file 41419_2021_3926_MOESM2_ESM.png]

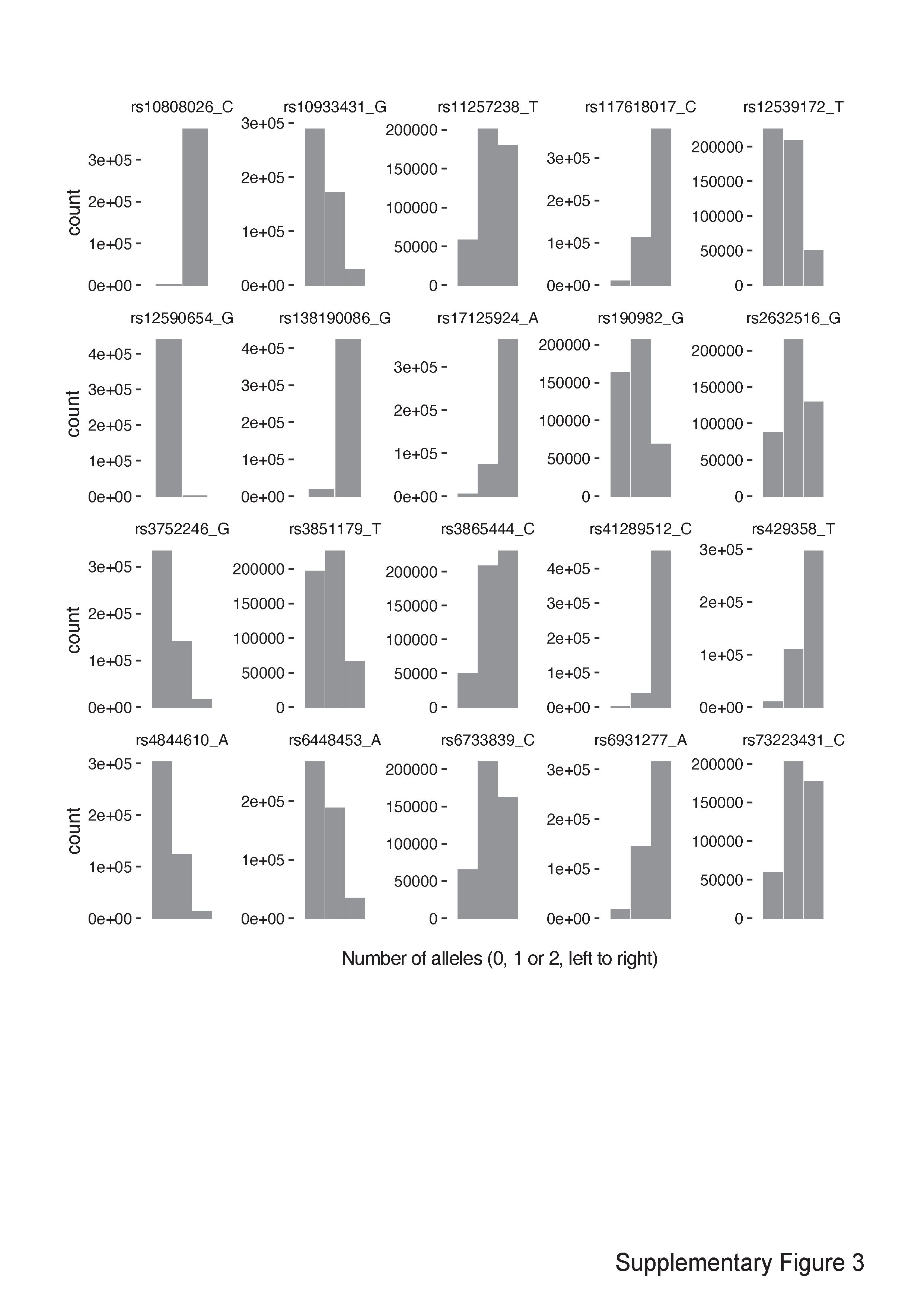

Supplement: Supplementary file 3 — Supplementary Figure 3 [file 41419_2021_3926_MOESM3_ESM.png]

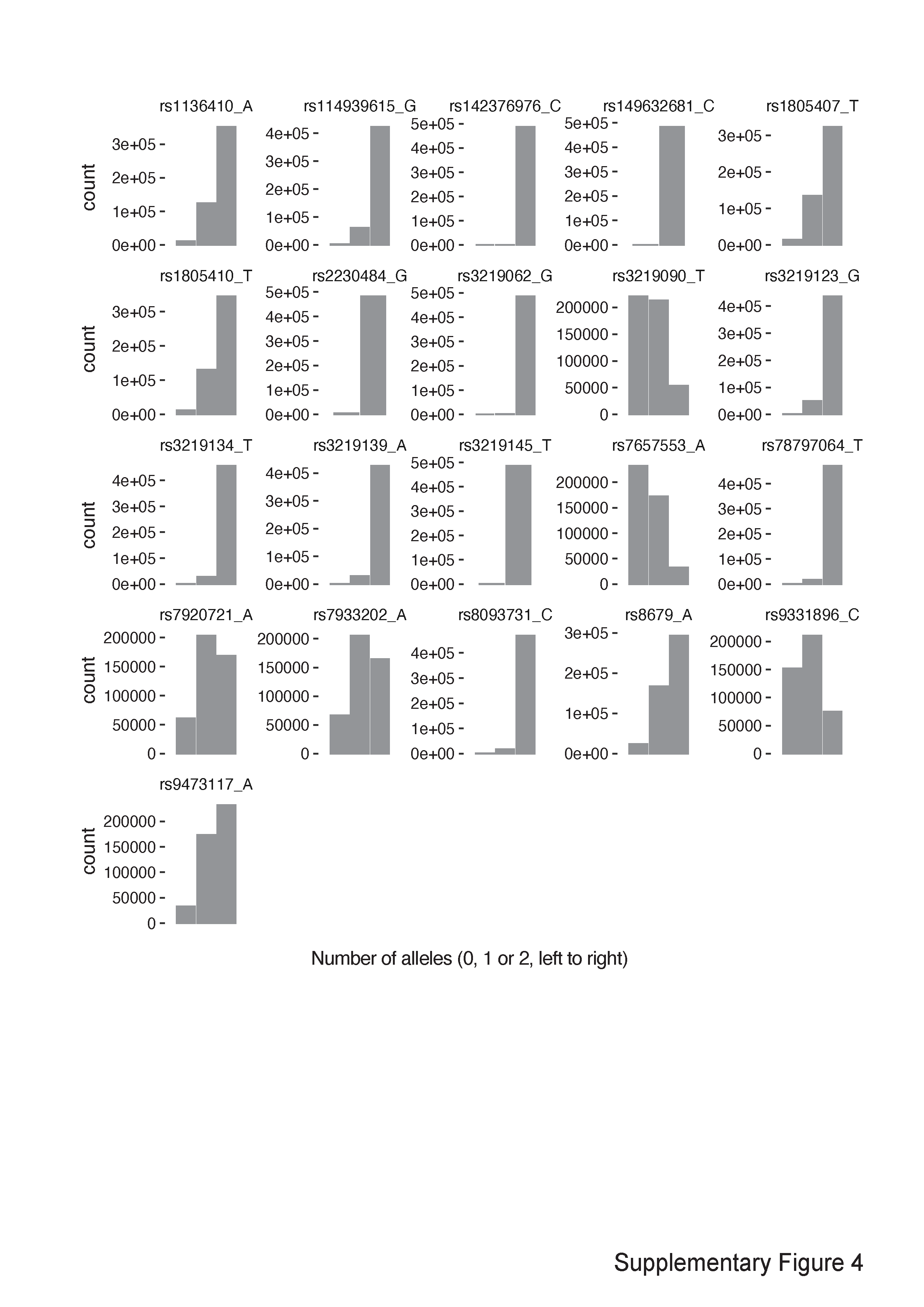

Supplement: Supplementary file 4 — Supplementary Figure 4 [file 41419_2021_3926_MOESM4_ESM.png]
